# Supplementary material for: Neuronal avalanches as a predictive biomarker for guiding tailored BCI training programs
Source: Imaging Neurosci (Camb). 2026 May 29;4:IMAG.a.1259. doi: 10.1162/IMAG.a.1259 (PMC13224313; doi:10.1162/IMAG.a.1259)
Supplement: Supplementary Material [file IMAG.a.1259_supp.pdf]

Supplementary Materials

Neuronal avalanches as a predictive biomarker for guiding tailored BCI training programs

Camilla Mannino[1*], Pierpaolo Sorrentino[2,3], Mario Chavez[1], Marie-Constance Corsi[1*]

[1]Sorbonne Université, Paris Brain Institute -ICM, CNRS, Inria, Inserm, AP-HP, Hôpital de la Pitié Salpêtrière, F-75013, Paris, France

[2]Institut de Neurosciences des Systèmes, Aix-Marseille Université, 13005 Marseille, France

[3]University of Sassari, Department of Biomedical Sciences, Viale San Pietro, 07100, Sassari, Italy

AVALANCHES' LENGTH : λ_{av}																				
	Global level analysis						***Local level analysis***													
	ANOVA test						***Friedman test***				***Wilcoxon test***									
	**Task-Effect**		**Learning Effect**		**Interaction Effect**		**Motor Imagery**		**Rest**		**Session 1**		**Session 2**		**Session 3**		**Session 4**			
Parameters' pair	*F-values*	*p-values*	*F-values*	*p-values*	*F-values*	*p-values*	*χ^2-values*	*p-values*	*χ^2-values*	*p- values*	*W-values*	*p-values*	*W-values*	*p-values*	*W-values*	*p-values*	*W-values*	*p-values*		
Θ_{av} : $\mu+\sigma$, λ_{min_av}: 5ms	1.450	0.243	1.208	0.3203	0.740	0.540	2.82	0.4202	2.82	0.420	71.0	0.216	103.0	0.956	56.0	0.070	68.0	0.177		
Θ_{av} : $\mu+\sigma$, λ_{min_av}: 50ms	1.308	0.259	1.192	0.314	0.752	0.535	2.82	0.420	1.98	0.577	69.0	0.189	81.0	0.388	59.0	0.090	76.0	0.294		
Θ_{av} : $\mu+\sigma$, λ_{min_av}: 80ms	1.216	0.275	1.190	0.323	0.766	0.516	5.34	0.149	3.06	0.390	68.0	0.177	65.0	0.143	61.0	0.106	80.0	0.368		
Θ_{av} : $\mu+2\sigma$, λ_{min_av}: 5ms	1.378	0.252	0.857	0.486	0.414	0.753	4.98	0.173	1.38	0.710	102.0	0.927	71.0	0.216	92.0	0.648	96.0	0.756		
Θ_{av} : $\mu+2\sigma$, λ_{min_av}: 50ms	0.179	0.680	0.850	0.482	0.397	0.770	1.68	0.641	1.02	0.796	78.0	0.330	83.0	0.430	78.0	0.330	79.0	0.349		
Θ_{av} : $\mu+2\sigma$, λ_{min_av}: 80ms	0.037	0.849	0.458	0.726	0.573	0.645	6.66	0.084	2.34	0.505	61.0	0.105	105.0	1.0	69.0	0.189	76.0	0.294		
Θ_{av} : $\mu+3\sigma$, λ_{min_av}: 5ms	0.021	0.886	0.288	0.840	1.990	0.121	4.5	0.212	7.38	0.061	77.0	0.312	85.0	0.475	73.0	0.246	53.0	0.053		
Θ_{av} : $\mu+3\sigma$, λ_{min_av}: 50ms	0.005	0.943	0.670	0.575	2.182	0.093	***9.66***	***0.022***	0.42	0.936	57.0	0.076	75.0	0.277	78.0	0.330	***34.0***	***0.006***		
Θ_{av} : $\mu+4\sigma$, λ_{min_av}: 5ms	3.406	0.067	0.628	0.597	1.523	0.205	5.1	0.165	1.2	0.753	76.0	0.294	88.0	0.546	75.0	0.277	56.0	0.070		
Θ_{av} : $\mu+5\sigma$, λ_{min_av}: 5ms	3.560	0.064	0.965	0.422	0.933	0.429	***8.7***	***0.034***	0.78	0.854	97.0	0.784	89.0	0.571	88.0	0.546	***52.0***	***0.048***		

Supplementary Materials, Table 1. Statistical results of all analysis for Avalanches' length λ_{av} over all possible parameters' combinations. In bold the significant values ($p < 0.05$)

ACTIVATIONS COUNT : α_{av}																		
	Global level analysis						***Local level analysis***											
	ANOVA test						***Friedman test***				***Wilcoxon test***							
	**Task-Effect**		**Learning Effect**		**Interaction Effect**		**Motor Imagery**		**Rest**		**Session 1**		**Session 2**		**Session 3**		**Session 4**	
Parameters' pair	*F-values*	*p-values*	*F-values*	*p-values*	*F-values*	*p-values*	*χ^2 values*	*p-values*	*χ^2-values*	*p- values*	*W-values*	*p-values*	*W-values*	*p-values*	*W-values*	*p-values*	*W-values*	*p-values*
Θ_{av} : $\mu+\sigma$, λ_{min_av}: 5ms	0.964	0.329	1.371	0.253	0.667	0.588	4.38	0.223	4.02	0.259	80	0.368	81	0.388	93	0.674	79	0.349
Θ_{av} : $\mu+\sigma$, λ_{min_av}: 50ms	0.904	0.348	1.468	0.229	0.676	0.576	5.28	0.152	5.46	0.141	79	0.349	79	0.349	96	0.756	82	0.409
Θ_{av} : $\mu+\sigma$, λ_{min_av}: 80ms	0.856	0.379	1.522	0.215	0.683	0.574	4.5	0.212	3.96	0.266	80	0.368	81	0.388	99	0.841	80	0.368
Θ_{av} : $\mu+2\sigma$, λ_{min_av}: 5ms	0.056	0.812	0.841	0.482	0.535	0.672	5.76	0.124	1.86	0.602	84	0.452	99	0.841	72	0.231	79	0.349
Θ_{av} : $\mu+2\sigma$, λ_{min_av}: 50ms	0.285	0.597	0.829	0.489	0.899	0.451	3.12	0.374	1.62	0.655	82	0.409	92	0.648	85	0.475	75	0.277
Θ_{av} : $\mu+2\sigma$, λ_{min_av}: 80ms	0.609	0.436	0.597	0.627	1.106	0.344	4.2	0.241	1.86	0.602	80	0.368	93	0.674	84	0.452	69	0.189
Θ_{av} : $\mu+3\sigma$, λ_{min_av}: 5ms	0.426	0.517	0.323	0.818	1.077	0.367	***12.78***	***0.005***	0.78	0.854	105	1.0	92	0.648	86	0.498	***47***	***0.030***
Θ_{av} : $\mu+3\sigma$, λ_{min_av}: 50ms	0.15	0.699	0.218	0.887	1.2	0.306	5.58	0.134	0.96	0.811	94	0.701	84	0.452	93	0.674	***52***	***0.048***
Θ_{av} : $\mu+4\sigma$, λ_{min_av}: 5ms	0.357	0.561	0.643	0.612	0.552	0.665	***9.42***	***0.024***	0.18	0.981	94	0.701	87	0.522	69	0.189	73	0.246
Θ_{av} : $\mu+5\sigma$, λ_{min_av}: 5ms	0.079	0.794	1.018	0.39	0.355	0.797	3.0	0.392	0.3	0.96	98	0.812	83	0.43	68	0.177	76	0.294

Supplementary Materials, Table 2. Statistical results of all analysis for Activations count α_{av} over all possible parameters' combinations. In bold the significant values ($p < 0.05$)

Repeated Correlation across sessions				
Parameters' pair	***ΔAVALANCHES' LENGTH $\Delta\lambda_{av}$***		***ΔACTIVATIONS COUNT $\Delta\alpha_{av}$***	
	r-values	*p-values*	*r-values*	*p-values*
Θ_{av} : $\mu+\sigma$, λ_{min_av}: 5ms	-0.01	0.905	0.004	0.976
Θ_{av} : $\mu+\sigma$, λ_{min_av}: 50ms	-0.01	0.917	0.01	0.937
Θ_{av} : $\mu+\sigma$, λ_{min_av}: 80ms	-0.02	0.943	0.01	0.917
Θ_{av} : $\mu+2\sigma$, λ_{min_av}: 5ms	-0.12	0.343	0.20	0.120
Θ_{av} : $\mu+2\sigma$, λ_{min_av}: 50ms	0.02	0.877	***0.29***	***0.022***
Θ_{av} : $\mu+2\sigma$, λ_{min_av}: 80ms	0.09	0.484	***0.26***	***0.040***
Θ_{av} : $\mu+3\sigma$, λ_{min_av}: 5ms	***0.27***	***0.033***	***0.28***	***0.027***
Θ_{av} : $\mu+3\sigma$, λ_{min_av}: 50ms	***0.39***	***0.002***	***0.28***	***0.028***
Θ_{av} : $\mu+4\sigma$, λ_{min_av}: 5ms	***0.48***	***0.0001***	0.20	0.122
Θ_{av} : $\mu+5\sigma$, λ_{min_av}: 5ms	***0.47***	***0.0002***	0.15	0.247

Supplementary Materials, Table 3. Repeated Correlation results across sessions between both features ($\Delta\lambda_{av}$, $\Delta\alpha_{av}$) and BCI-scores over all possible parameters' combinations. In bold significant correlations ($p < 0.05$)

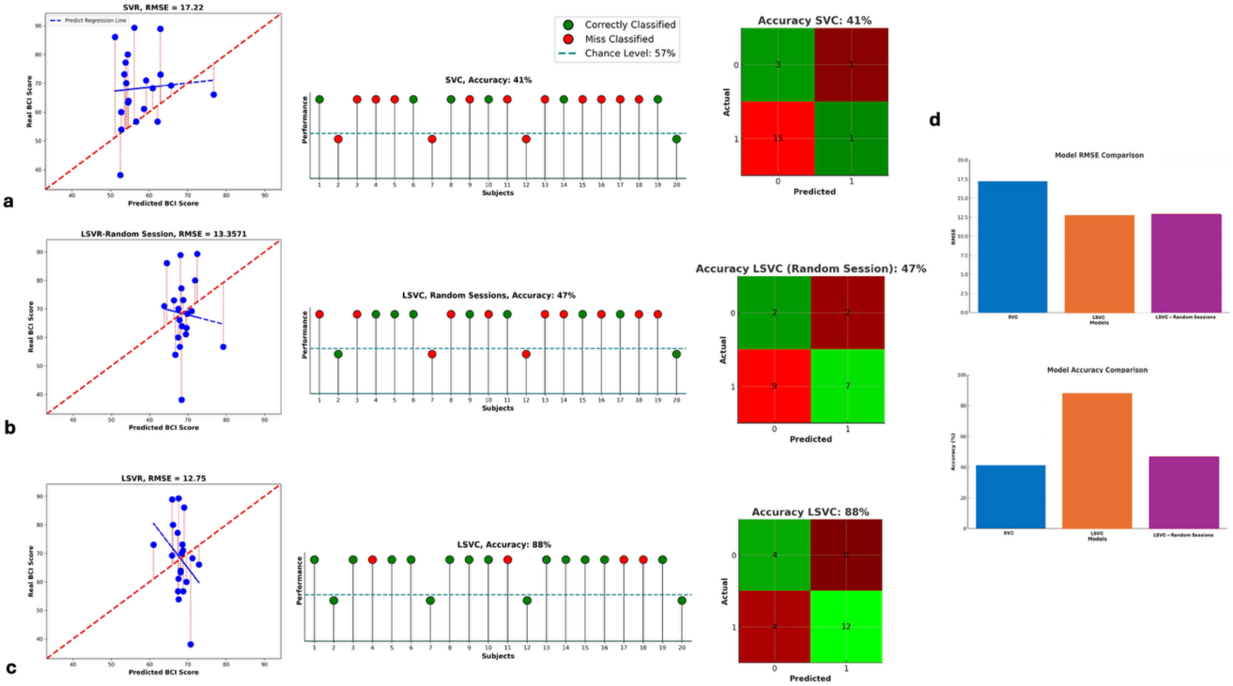

Supplementary Materials, Figure 1. Predictive Model Results.

a. Predictive results using Support Vector Regression (SVR) (left panel) and Support Vector Classification (SVC) (right panel) models.

b. Predictive results using Longitudinal Support Vector Regression (LSVR) (left panel) and Longitudinal Support Vector Classification (LSVC) (right panel) models with sessions in random order.

c. Predictive results using Longitudinal Support Vector Regression (LSVR) (left panel) and Longitudinal Support Vector Classification (LSVC) (right panel) models.

> **Left Panel:** Regression Results: Each point represents a subject. Red dashed lines indicate the prediction error between actual and predicted values. The bold red line represents the optimal prediction trend, while the blue lines show individual predicted trends.
>
> **Right Panel:** Classification Results and Confusion Matrix**:** Each ball represents a subject. The dashed horizontal line denotes the classification threshold—subjects above this line are predicted to have control. Green balls indicate correct predictions, while red balls represent misclassifications. In the confusion matrix, green cells show correctly classified subjects, and red cells indicate misclassifications. The intensity of each cell's color corresponds to the number of subjects in that category.

d. Comparison of different models.

> **Top:** Comparison of the Root Mean Square Error (RMSE) obtained using different models.
>
> **Bottom:** Comparison of the accuracy performance obtained using different models. SVC (blue), LSVC (orange), and LSVC with random sessions (purple).

All these plots are generated using θ_{av} $\mu + 3\sigma$ and λ_{min_av} : 50ms, and the best-coupled parameters for prediction.

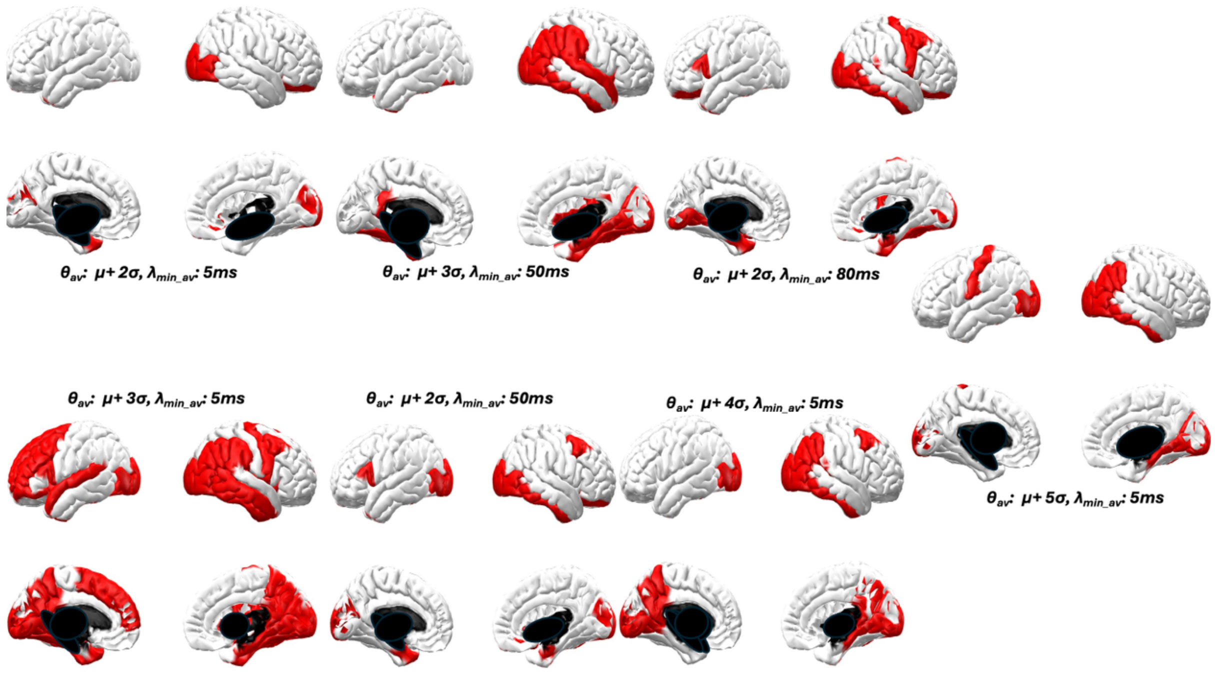

Supplementary Materials, Figure 2. Selected ROIs set for each pair of parameters. In red the ROIs that show significance after ANOVA across all the four sessions of t-values between Motor Imagery and resting condition.

AVALANCHES' LENGTH : λ_{av}																			
	Global level analysis						*Local level analysis*												
	ANOVA test						*Friedman test*				*Wilcoxon test*								
	**Task-Effect**		**Learning Effect**		**Interaction Effect**		**Motor Imagery**		**Rest**		**Session 1**		**Session 2**		**Session 3**		**Session 4**		
Parameters' pair	*F-values*	*p-values*	*F-values*	*p-values*	*F-values*	*p-values*	*χ^2-values*	*p-values*	*χ^2-values*	*p- values*	*W-values*	*p-values*	*W-values*	*p-values*	*W-values*	*p-values*	*W-values*	*p-values*	
Θ_{av} : $\mu+2\sigma$, λ_{min_av}: 5ms	*3.255*	*0.076*	***3.189***	***0.024***	2.075	0.111	2.4	0.494	***7.98***	***0.046***	70	0.202	78	0.330	75	0.277	***24***	***0.001***	
Θ_{av} : $\mu+2\sigma$, λ_{min_av}: 50ms	2.141	0.139	0.210	0.892	2.221	0.086	***10.5***	***0.015***	1.5	0.682	83	0.43	89	0.571	97	0.784	***12***	***0.0001***	
Θ_{av} : $\mu+2\sigma$, λ_{min_av}: 80ms	0.003	0.958	0.818	0.521	0.930	0.457	5.46	0.141	0.72	0.869	90	0.596	105	1.0	102	0.927	69	0.189	
Θ_{av} : $\mu+3\sigma$, λ_{min_av}: 5ms	1.771	0.184	0.577	0.632	2.413	0.070	2.7	0.440	1.5	0.682	64	0.133	84	0.452	70	0.202	***44***	***0.022***	
Θ_{av} : $\mu+3\sigma$, λ_{min_av}: 50ms	0.287	0.592	0.793	0.512	1.143	0.331	***9.72***	***0.021***	1.62	0.655	98	0.812	89	0.571	101	0.898	***46***	***0.027***	
Θ_{av} : $\mu+4\sigma$, λ_{min_av}: 5ms	***5.903***	***0.015***	1.184	0.318	0.652	0.582	3.42	0.331	0.9	0.825	105	1.0	84	0.452	69	0.189	*54*	*0.058*	
Θ_{av} : $\mu+5\sigma$, λ_{min_av}: 5ms	***4.412***	***0.034***	2.201	0.090	1.198	0.312	***11.7***	***0.009***	5.82	0.121	87	0.522	76	0.294	60	0.097	***49***	***0.036***	

Supplementary Materials, Table 4. Statistical results of all analysis for Avalanches' length λ_{av} over all possible parameters' combinations using a selected set of ROIs. In bold the significant values ($p < 0.05$)

ACTIVATIONS COUNT : α_{av}																				
	Global level analysis						*Local level analysis*													
	ANOVA test						*Friedman test*				*Wilcoxon test*									
	**Task-Effect**		**Learning Effect**		**Interaction Effect**		**Motor Imagery**		**Rest**		**Session 1**		**Session 2**		**Session 3**		**Session 4**			
Parameters' pair	*F-values*	*p-values*	*F-values*	*p-values*	*F-values*	*p-values*	*χ^2-values*	*p-values*	*χ^2-values*	*p-values*	*W-values*	*p-values*	*W-values*	*p-values*	*W-values*	*p-values*	*W-values*	*p-values*		
Θ_{av} : μ+2σ, λ_{min_av}: 5ms	***4.014***	***0.045***	1.477	0.222	1.791	0.145	***8.22***	***0.042***	0.96	0.811	99	0.841	94	0.701	97	0.784	***38***	***0.011***		
Θ_{av} : μ+2σ, λ_{min_av}: 50ms	0.969	0.333	0.056	0.984	0.958	0.423	5.82	0.121	2.04	0.564	92	0.648	79	0.349	85	0.475	***31***	***0.004***		
Θ_{av} : μ+2σ, λ_{min_av}: 80ms	0.008	0.939	0.376	0.818	0.628	0.644	2.46	0.483	0.9	0.825	90	0.596	103	0.956	80	0.368	78	0.33		
Θ_{av} : μ+3σ, λ_{min_av}: 5ms	1.185	0.285	0.325	0.815	1.224	0.303	10.68	0.014	0.24	0.971	97	0.784	92	0.648	84	0.452	***48***	***0.033***		
Θ_{av} : μ+3σ, λ_{min_av}: 50ms	0.516	0.485	0.722	0.555	0.906	0.456	*7.5*	*0.058*	1.56	0.669	88	0.546	75	0.277	87	0.522	***47***	***0.030***		
Θ_{av} : μ+4σ, λ_{min_av}: 5ms	3.155	0.076	0.604	0.625	0.642	0.597	5.94	0.115	1.56	0.669	103	0.956	96	0.756	90	0.596	61	0.105		
Θ_{av} : μ+5σ, λ_{min_av}: 5ms	1.372	0.263	1.988	0.091	0.879	0.472	6.36	0.095	1.32	0.724	101	0.898	99	0.841	68	0.177	61	0.105		

Supplementary Materials, Table 5. Statistical results of all analysis for Activations count α_{av} over all possible parameters' combinations using a selected set of ROIs. In bold the significant values ($p < 0.05$)

Repeated Correlation across sessions				
Parameters' pair	***ΔAVALANCHES' LENGTH $\Delta\lambda_{av}$***		***ΔACTIVATIONS COUNT $\Delta\alpha_{av}$***	
	r-values	*p-values*	*r-values*	*p-values*
Θ_{av} : μ+2σ, λ_{min_av}: 5ms	***0.47***	***0.0001***	***0.33***	***0.010***
Θ_{av} : μ+2σ, λ_{min_av}: 50ms	***0.32***	***0.011***	***0.26***	***0.046***
Θ_{av} : μ+2σ, λ_{min_av}: 80ms	0.11	0.411	0.17	0.194
Θ_{av} : μ+3σ, λ_{min_av}: 5ms	***0.44***	***0.0004***	0.23	0.072
Θ_{av} : μ+3σ, λ_{min_av}: 50ms	0.10	0.451	0.08	0.557
Θ_{av} : μ+4σ, λ_{min_av}: 5ms	***0.31***	***0.014***	0.20	0.121
Θ_{av} : μ+5σ, λ_{min_av}: 5ms	***0.25***	***0.047***	0.17	0.181

Supplementary Materials, Table 6. Repeated Correlation results across sessions between both features ($\Delta\lambda_{av}$, $\Delta\alpha_{av}$) and BCI-scores over all possible parameters' combinations using a selected set of ROIs. *In bold significant correlations ($p < 0.05$)*

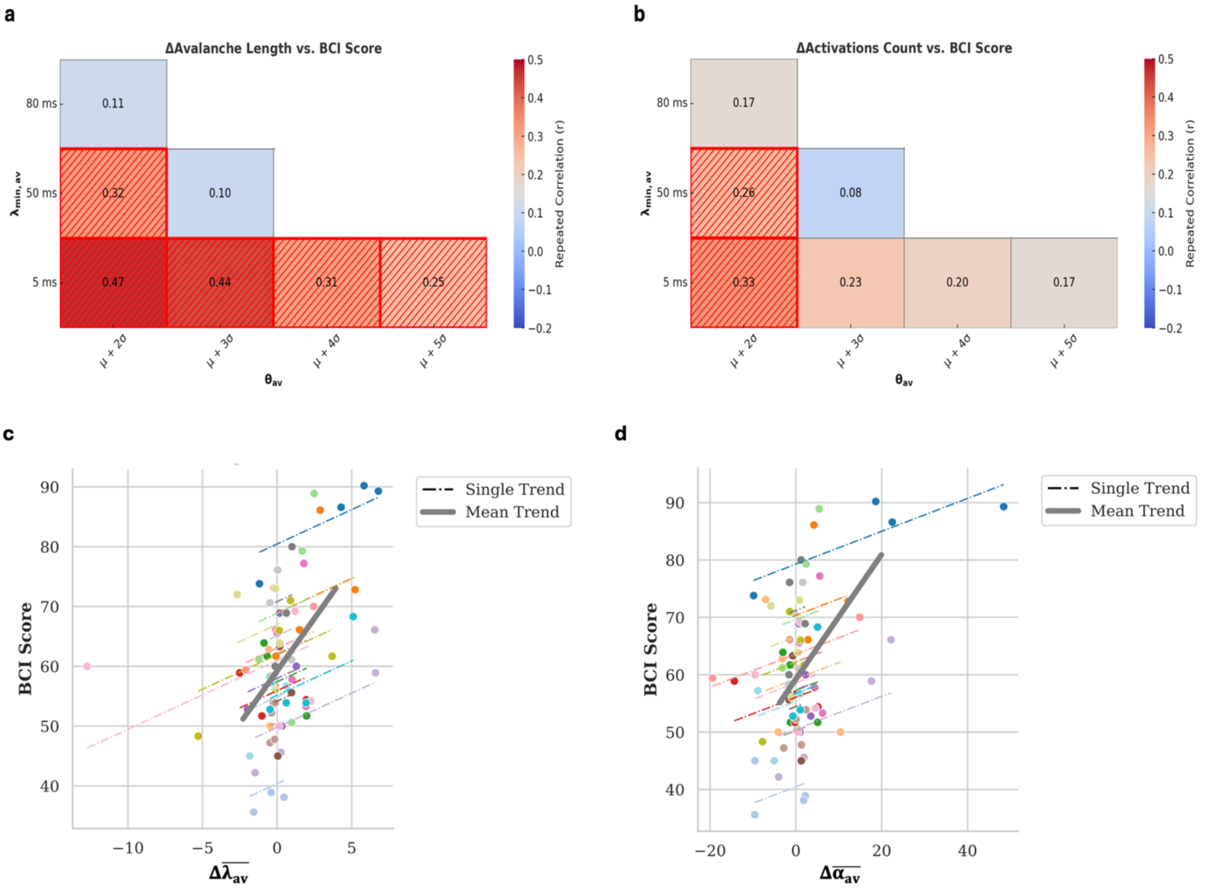

Supplementary Materials, Figure 3. Repeated correlation and trends across different sessions between BCI score and features changes over a selected set of ROIs. ***a.*** Mean difference of avalanches' length ($\Delta\lambda_{av}$) and ***b.*** Mean difference activation ($\Delta\alpha_{av}$) across all tested pairs of parameters (θ_{av}, λ_{min_av}). Significant correlations ($p<0.05$) are highlighted using different textures. ***c.*** Repeated correlations trend across different sessions between BCI-score and $\Delta\lambda_{av}$ and ***d.*** between $\Delta\alpha_{av}$ and BCI-score. Each coloured dashed line corresponds to one subject while the grey bold line identifies the trend across all the subjects. The pairs of parameters (θ_{av}, λ_{min_av}) used in ***(c)*** and ***(d)*** were those that achieve the best prediction performance: θ_{av}: $\mu+2\sigma$, λ_{min_av} : 50ms.

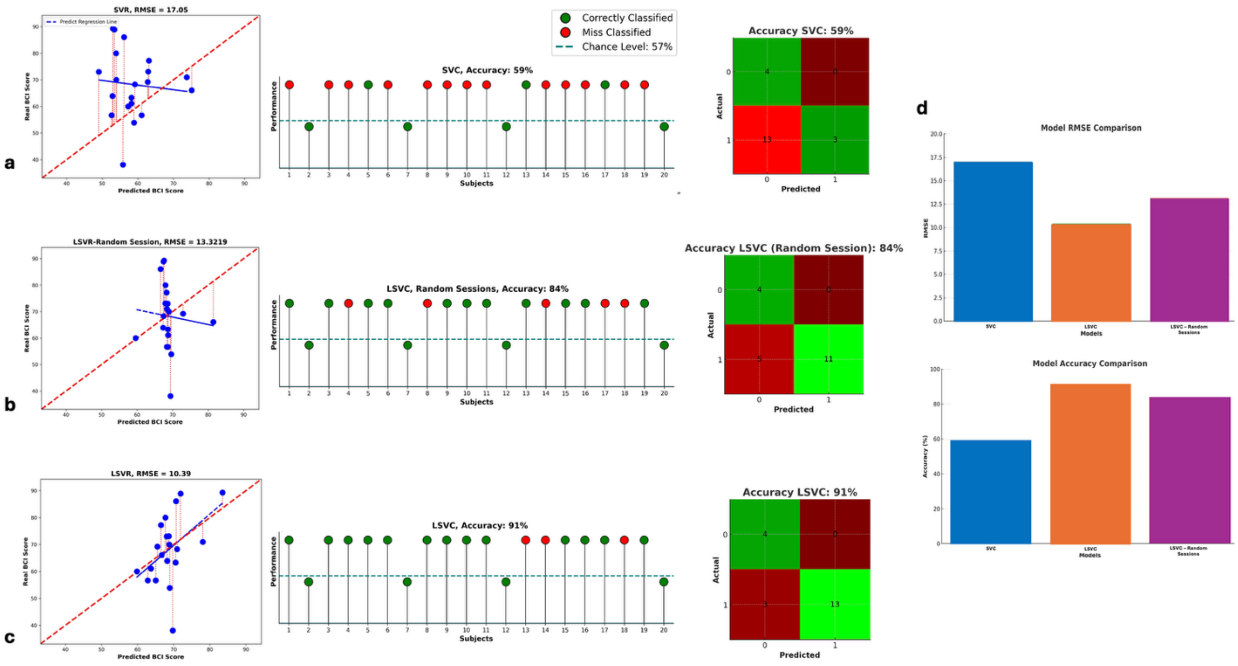

Supplementary Materials, Figure 4: Predictive Model Results over a selected set of ROIs.

a. Predictive results using Support Vector Regression (SVR) (left panel) and Support Vector Classification (SVC) (right panel) models.

b. Predictive results using Longitudinal Support Vector Regression (LSVR) (left panel) and Longitudinal Support Vector Classification (LSVC) (right panel) models with sessions in random order.

c. Predictive results using Longitudinal Support Vector Regression (LSVR) (left panel) and Longitudinal Support Vector Classification (LSVC) (right panel) models.

Left Panel: Regression Results: Each point represents a subject. Red dashed lines indicate the prediction error between actual and predicted values. The bold red line represents the optimal prediction trend, while the blue lines show individual predicted trends.

Right Panel: Classification Results and Confusion Matrix**:** Each ball represents a subject. The dashed horizontal line denotes the classification threshold—subjects above this line are predicted to have control. Green balls indicate correct predictions, while red balls represent misclassifications. In the confusion matrix, green cells show correctly classified subjects, and red cells indicate misclassifications. The intensity of each cell's color corresponds to the number of subjects in that category.

d. Comparison of different models.

Top: Comparison of the Root Mean Square Error (RMSE) obtained using different models.

Bottom: Comparison of the accuracy performance obtained using different models. SVC (blue), LSVC (orange), and LSVC with random sessions (purple).

All these plots are generated using θ_{av} $\mu + 2\sigma$ and λ_{min_av} : 50ms, and the best-coupled parameters for prediction.

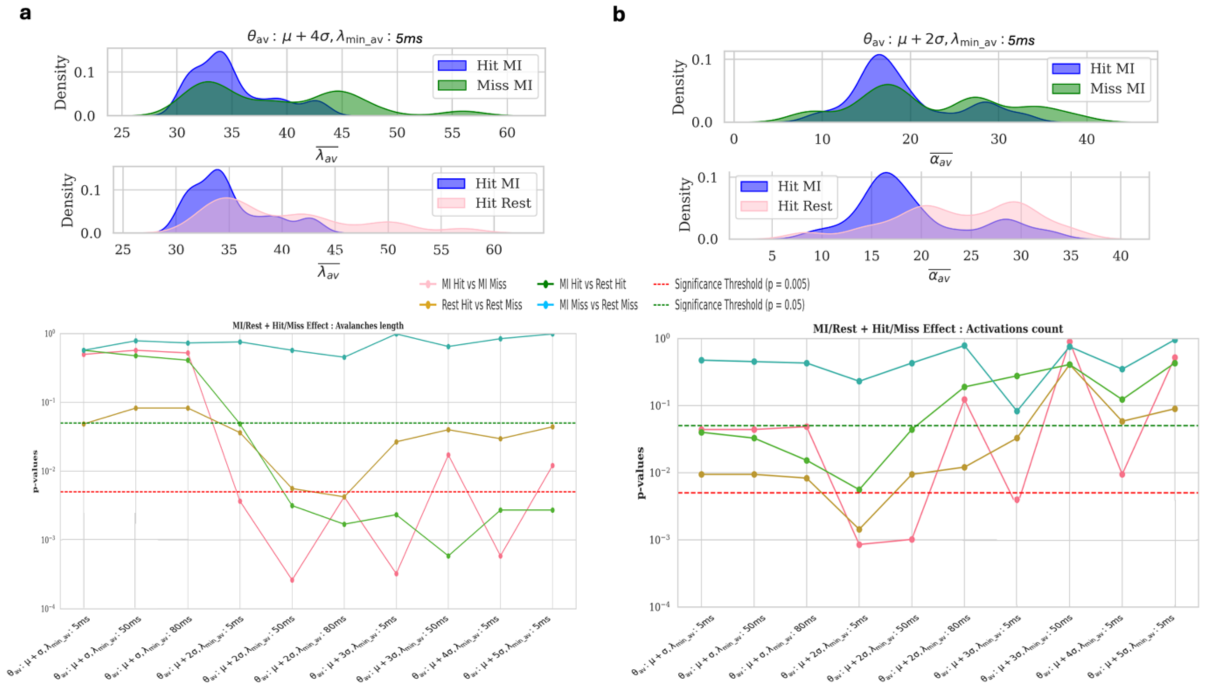

Supplementary Materials, Figure 5. Analysis of Mean Avalanche Length and Activations in Hit vs. Miss Trials.

a. Mean Avalanche Length in Hit Trials vs. Miss Trials:

> **Top:** Probability density functions representing: i) Hit Motor Imagery (MI) trials (in blue) vs. Hit Rest trials (in pink) on the left ; ii) Hit MI trials (in blue) vs. Miss MI trials (in green) on the right.
>
> **Bottom:** Trends of p-values from pairwise comparisons of mean avalanche length across brain states (MI vs. Rest) and conditions (Hit vs. Miss) across possible coupled parameters.

Dashed horizontal lines indicate significance thresholds (green: $p < 0.05$: red: $p < 0.001$, Bonferroni-corrected).

b. Activations in Hit Trials vs. Miss Trials:

> **Top:** Probability density functions representing: i) Hit Motor Imagery (MI) trials (in blue) vs. Hit Rest trials (in pink) on the left ; ii) Hit MI trials (in blue) vs. Miss MI trials (in green) on the right.
>
> **Bottom:** Trends of p-values from pairwise comparisons of activation counts across brain states (MI vs. Rest) and conditions (Hit vs. Miss) across possible coupled parameters.

Dashed horizontal lines indicate significance thresholds (green:$p < 0.05$, red: $p < 0.001$, Bonferroni-corrected).

All these analyses are performed only on the last training session.

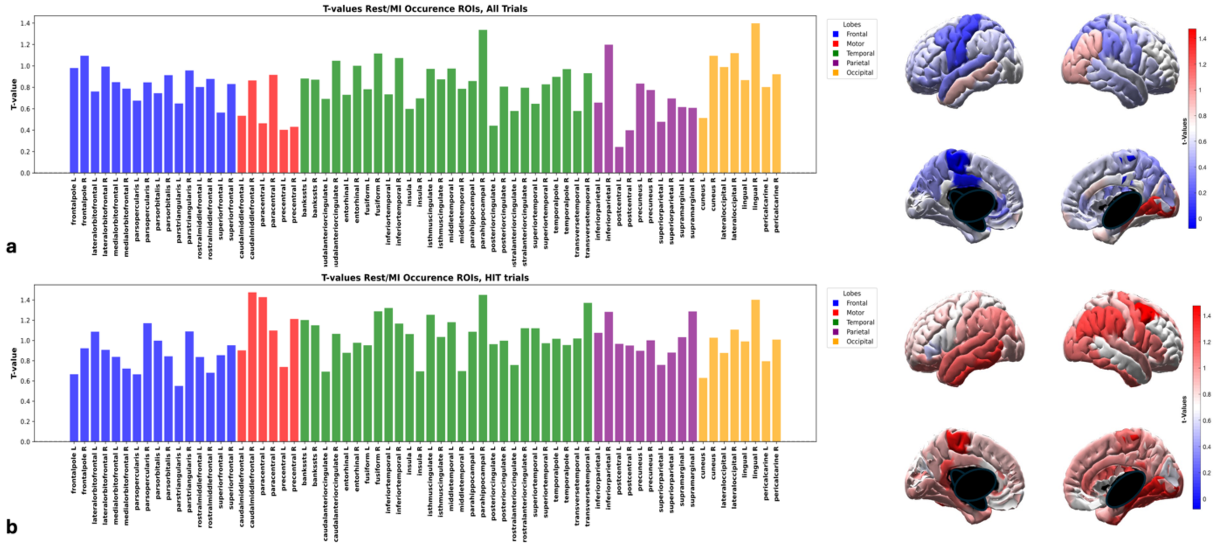

Supplementary Materials, Figure 6. t-values Comparing Rest and Motor Imagery (MI) Conditions.
a. t-values calculated for the occurrence of each specific Region of Interest (ROI) across different trials, considering all trials.
b. t-values calculated for the occurrence of each specific ROI across different trials, considering only those trials in which the subject successfully controlled the device.
In the brain plots, the color map reflects the magnitude of the t-values, while in the bar plots, it corresponds to the height of each bar. Brain regions are color-coded as follows: red for the motor cortex, green for the temporal lobe, yellow for the occipital lobe, blue for the frontal lobe, purple for the parietal lobe. All these analyses were performed only on the last training session and one specific parameter combination: θ_{av}: $\mu + 4\sigma$, λ_{min_av}: 2.
